# LIVE-CELL FLUORESCENCE MICROSCOPY OF HSV-1 CELLULAR EGRESS BY EXOCYTOSIS

**DOI:** 10.1101/2023.02.27.530373

**Published:** 2023-02-28

**Authors:** Melissa H. Bergeman, Michaella Q. Hernandez, Jenna Diefenderfer, Jake A. Drewes, Kimberly Velarde, Wesley M. Tierney, Ian B. Hogue

**Affiliations:** Biodesign Center for Immunotherapy, Vaccines, and Virotherapy & School of Life Sciences, Arizona State University, Tempe, Arizona, United States

## Abstract

The human pathogen Herpes Simplex Virus 1 (HSV-1) produces a lifelong infection in the majority of the world’s population. While the generalities of alpha herpesvirus assembly and egress pathways are known, the precise molecular and spatiotemporal details remain unclear. In order to study this aspect of HSV-1 infection, we engineered a recombinant HSV-1 strain expressing a pH-sensitive reporter, gM-pHluorin. Using a variety of fluorescent microscopy modalities, we can detect individual virus particles undergoing intracellular transport and exocytosis at the plasma membrane. We show that particles exit from epithelial cells individually, not bulk release of many particles at once, as has been reported for other viruses. In multiple cell types, HSV-1 particles accumulate in clusters at the corners, edges, and cell-cell contacts. We show that this clustering effect is the result of individual particles undergoing exocytosis at preferential sites. We also show that the viral membrane proteins gE, gI, and US9, which have important functions in intracellular transport in neurons, have a subtle effect on preferential egress and clustering in non-neuronal cells. Importantly, by comparing HSV-1 to a related alpha herpesvirus, pseudorabies virus, we show that this preferential exocytosis and clustering effect is cell type-dependent, not virus dependent.

## Introduction

The human pathogen Herpes Simplex Virus type 1 (HSV-1) is a member of the alpha herpesvirus sub-family, which includes several endemic human pathogens, economically-important veterinary pathogens, and zoonotic pathogens that can be severely neuroinvasive. The generalities of alpha herpesvirus assembly and egress pathways are known, but the spatiotemporal details remain unclear. Viral DNA replication and packaging occurs in the nucleus, nuclear egress occurs by transient envelopment/de-envelopment at the nuclear membranes [1–3], and secondary envelopment occurs on intracellular membranes derived from the secretory and endocytic pathways [4–11]. Following secondary envelopment, the secretory organelle containing the enveloped virion traffics to the plasma membrane, predominantly using microtubule motors, where it is released by exocytosis [12–17]. However, the precise molecular details, virus-host interactions, and dynamics of this process are not well understood.

While fluorescence microscopy has been widely used to determine the relationships between viral proteins and cellular markers, it lacks the spatial resolution to determine the precise assembly state of the virion. Electron microscopy has also provided many insightful results, but this technique is confounded by difficulties in sample preparation, small sample sizes, and the fact that samples are fixed and static [18–21]. This last constraint is of particular concern as it does not offer insights into the dynamic aspects of the viral replication cycle in infected cells. To overcome some of these limitations, we previously developed a live-cell fluorescence microscopy method to study exocytosis of the important veterinary and zoonotic virus, Pseudorabies Virus (PRV; suid alphaherpesvirus 1) [16, 17]. In these studies, we showed that PRV particles exit from infected cells by exocytosis using cellular secretory mechanisms, are mainly released as single particles from individual secretory vesicles, and the spatial distribution viral exocytosis is largely uniform across the adherent cell surface (in PK15 cells, a non-polarized porcine kidney epithelial cell line). However, other studies focusing on HSV-1 showed that viral proteins and particles accumulate at preferential locations, which the authors inferred was the result of viral exocytosis at preferential “hot spots” in Vero cells, a non-polarized African green monkey kidney epithelial cell line [22–25].

In other viruses, a variety of egress modes have been observed: The beta herpesvirus human cytomegalovirus (HCMV) was shown to exit by bulk release – exocytosis of many particles from a larger organelle – in human foreskin fibroblast (HFF-1) cells [26]. Both flaviviruses and coronaviruses have been observed by electron microscopy to accumulate large numbers of virus particles in large intracellular organelles, but it is unclear whether these large organelles mediate bulk release or if there are subsequent intracellular sorting steps to release single virions from individual exocytosis events [27, 28]. In retroviruses, HIV-1 has been observed to assemble and exit preferentially at the trailing uropod of polarized T cells [29, 30], and human T-lymphotropic virus (HTLV) forms large accumulations of virions and extracellular matrix (termed “viral biofilms”) on the cell surface that may promote more efficient cell-cell spread [31–34]. Thus, the relationship between exocytosis (single particles in individual secretory vesicles, versus bulk release of many particles from a larger organelle) and accumulation at preferential locations on the cell surface following exocytosis varies according to the particular virus and cell type, but is likely important for subsequent cell-cell spread.

**Figure 1.**
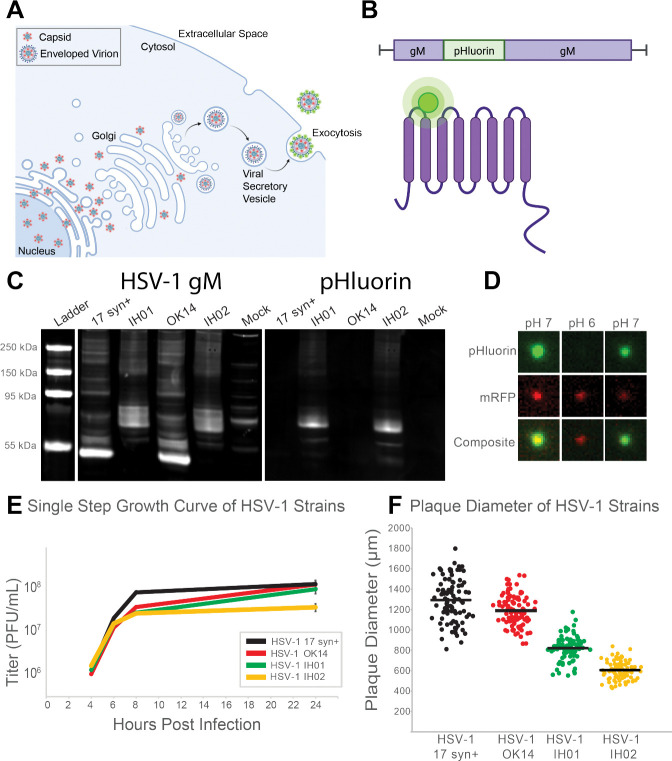
HSV-1 Egress and gM-pHluorin Insert **A.** Schematic of HSV-1 egress from infected cells. Nonenveloped virus capsids (red) exit the nucleus and traffic to the site of secondary envelopment. Following secondary envelopment, virions are transported to the plasma membrane by acidic secretory organelles. pHluorin (green) dequenches upon exocytosis at the plasma membrane. **B.** Schematic of gM-pHluorin, with the pHluorin moiety (green) inserted into the first extravirion loop of gM (purple). **C.** Western blot detecting HSV-1 gM and pHluorin. Vero cells were infected with HSV-1 17syn^+^, IH01, OK14, and IH02, or mock-infected. Blots were probed with primary antibodies detecting gM and pHluorin, and imaged using fluorescent secondary antibodies to detect HSV-1 gM and pHluorin simultaneously. **D.** gM-pHluorin is incorporated into virus particles, and exhibits reversibly pH-sensitive fluorescence. Freshly-prepared supernatants from Vero cells infected with HSV-1 IH02 were spotted onto glass bottom dishes. Particles were imaged at pH ~7, pH ~6, and pH ~7. A representative virus particle is shown with gM-pHluorin and mRFP-VP26. Images represent 3.6×3.6 *μ*m. **E.** Single step growth curve. Vero cells were infected with HSV-1 17syn^+^, OK14 , IH01, or IH02 in triplicate, and harvested at 4, 6, 8, and 24 hpi. Samples were titered by serial-dilution plaque assay. **F.** Plaque size measurements. At 4 days post-infection, virus plaques were imaged, and the zone of clearance diameter was measured in Fiji software and used to calculate mean plaque sizes (n=100).

In the present study, we have extended our previous work on PRV to visualize the exocytosis of HSV-1 particles via Total Internal Reflection Fluorescence (TIRF) microscopy. We engineered a recombinant strain of HSV-1 which expresses the fluorescent protein superecliptic pHluorin on an extravirion loop of the multipass transmembrane glycoprotein M (gM). A variant of GFP, pHluorin was developed as a means to image secretory vesicle exocytosis in a variety of cell types, including neurons [35, 36]. Following secondary envelopment, pHluorin is quenched in the acidic lumen of secretory vesicles (pH of 5.2–5.7) [36, 37]. When the secretory vesicle fuses with the plasma membrane to release the virus particle to the extracellular medium (pH ~7.5), pHluorin is dequenched and becomes brightly fluorescent, allowing the unambiguous identification of individual viral exocytosis events [16, 17, 37, 38] (Figure 1A). Using this technique, we show that HSV-1 exits from infected cells by exocytosis of individual virus particles, not bulk release of many virions at once. In some cell types, viral exocytosis occurs at preferential plasma membrane sites, leading to the gradual accumulation of large clusters of virus particles, but we show that this phenomenon is cell-type dependent. Consistent with previous reports [22], mutations in viral membrane proteins gE, gI, and US9 had modest effects, but were not essential for preferential viral egress and accumulation into clusters.

## Results

### Insertion of pHluorin into gM.

To produce the recombinant strain HSV-1 IH01, we inserted the pHluorin coding sequence into the gM (UL10) gene in the HSV-1 genome by homologous recombination between a synthesized shuttle plasmid and purified HSV-1 DNA. The construct was designed to insert the pHluorin moiety into the first extravirion loop of gM (Figure 1B). A second recombinant, HSV-1 IH02, expressing gM-pHluorin and an mRFP-VP26 capsid tag, was produced by co-infecting HSV-1 IH01 and HSV-1 OK14 [39], and purifying two-color plaques.

We confirmed the correct recombination occurred by PCR amplification and Sanger sequencing (data not shown). We also confirmed expression of gM-pHluorin by western blot of infected cell lysates, probing with anti-gM and anti-GFP antibodies simultaneously (Figure 1C). Viral membrane proteins frequently produce complex banding patterns due to differences in glycosylation and aggregation of these highly hydrophobic proteins during sample prep [40, 41]. Cells infected with parental strains HSV-1 17syn^+^ and OK14 produced major gM-immunoreactive bands near the predicted 51 kDa of native gM, whereas cells infected with the recombinant HSV-1 IH01 and IH02 strains produced bands that are immunoreactive to both gM and GFP antibodies, and shifted ~30 kDa, consistent with the predicted gM-pHluorin fusion (Figure 1C).

### gM-pHluorin Labels Virus Particles and Exhibits pH-Sensitive Fluorescence.

To determine whether gM-pHluorin is incorporated into individual virus particles, we spotted ~100*μ*l of freshly-prepared infected cell supernatants onto a glass coverslip, and imaged by fluorescence microscopy (Figure 1D). To measure the pH sensitivity of the gM-pHluorin fluorescence, we added an excess of PBS buffer at pH ~6 followed by an excess of PBS buffer at pH ~7. gM-pHluorin incorporated into virus particles exhibited reversible pH-dependent green fluorescence, whereas the mRFP-VP26 capsid tag exhibited a non-pH-sensitive reduction in fluorescence due to photobleaching (Figure 1D).

### Virus Replication.

To determine whether the recombinant HSV-1 IH01 and IH02 strains replicate comparably to the parental viruses, we performed single-step growth curves (Figure 1E) and measured plaque size (Figure 1F) on Vero cell monolayers. Compared to the parental HSV-1 17syn^+^ strain, HSV-1 OK14, IH01, and IH02 exhibited a modest delay in replication at 8 hours post-infection (hpi). By 24 hpi, HSV-1 OK14 and IH01 caught up to 17syn^+^, but HSV-1 IH02 exhibited a modest <1 log defect (Figure 1E). These data suggest that the gM-pHluorin and mRFP-VP26 fluorescent protein fusions result in a small reduction in viral replication, and these defects are additive in the HSV-1 IH02 recombinant that expresses both. Consistent with these results, plaque sizes of the HSV-1 OK14 and IH01 were also reduced, and these defects were additive in the HSV-1 IH02 recombinant (Figure 1F).

### Live-Cell Fluorescence Microscopy of Virus Particle Exocytosis.

**Figure 2.**
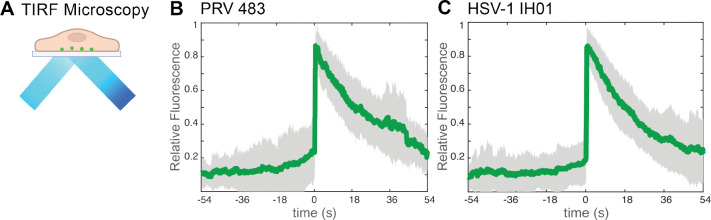
TIRF Microscopy of HSV-1 Exocytosis **A.** Schematic of TIRF microscopy. The excitation laser excites fluorescent molecules near the coverslip, and excludes out-of-focus fluorescence from deeper in the cell. **B-C.** Relative fluorescence intensity of gM-pHluorin before, during, and after exocytosis of individual virus particles. Green line represents mean fluorescence and gray shading indicates standard deviation. **B.** PRV 483 exocytosis events in PK15 cells, at 4–5 hpi (n=31). C. HSV-1 IH01 exocytosis events in Vero cells, at 5–6 hpi (n=67).

To investigate virus particle exocytosis, we infected Vero cells with HSV-1 IH01 at a high multiplicity of infection (MOI) to roughly synchronize viral infection, and imaged by live-cell fluorescence microscopy at approximately 5–6 hpi. This time point represents the earliest production of viral progeny, and prior to the onset of cytopathic effects. To compare to our previous studies of PRV [16], we also infected PK15 cells with PRV 483, which expresses orthologous gM-pHluorin and mRFP-VP26 fusions. We identified productively infected cells by imaging in widefield fluorescence mode to detect mRFP-VP26 red fluorescence in the nucleus and gM-pHluorin green fluorescence on the plasma membrane and in intracellular membranes. We then acquired timelapse movies in TIRF microscopy mode, which excludes out-of-focus fluorescence and emphasizes particle dynamics near the adherent cell surface (Figure 2A).

As previously reported with PRV, viral exocytosis events are characterized by the sudden (<90 ms) appearance of green gM-pHluorin fluorescence, which then remains puntate and mostly immobile during the time of imaging (>2–3 min) [16, 17]. To quantify this process over many exocytosis events, we measured the relative fluorescence intensity at exocytosis sites for 54 sec before and after each exocytosis event, aligned all data series to a common time=0, and calculated the ensemble average over many events (Figure 2B–C). Prior to exocytosis at time=0, the relative gM-pHluorin fluorescence remains low, consistent with pHluorin quenching in the acidic lumen of the viral secretory vesicle. At the moment of exocytosis, gM-pHluorin fluorescence increases suddenly due to dequenching at extracellular pH. Finally, the fluorescence decays gradually, which represents a combination of: 1. diffusion of gM-pHluorin that is incorporated into the vesicle membrane; 2. occasional movement of the cell or virus particle after exocytosis; 3. photobleaching. These data are consistent with our previous studies of PRV exocytosis [16, 17], validating that this approach works for HSV-1.

### HSV-1 Particles Accumulate at Preferential Exocytosis Sites.

Previous studies showed that HSV-1 structural proteins and particles accumulate in large clusters at the adherent corners and edges of Vero cells, and cell-cell junctions in epithelial cells [22–25]. However, based on static fluorescence and electron microscopy images, it is unclear if virus particles gradually accumulate in these clusters due to preferential exocytosis at these sites, if large clusters are deposited at once due to bulk release (as recently observed with HCMV [26]), or if virus particles accumulate in clusters later in infection due to cell movement and rounding associated with cytopathic effects. Previously, we did not observe preferential exocytosis sites or large clusters of virus particles with PRV in PK15 cells [16, 17], so it was unclear whether this represents a difference between viruses or a cell-type difference.

**Figure 3.**
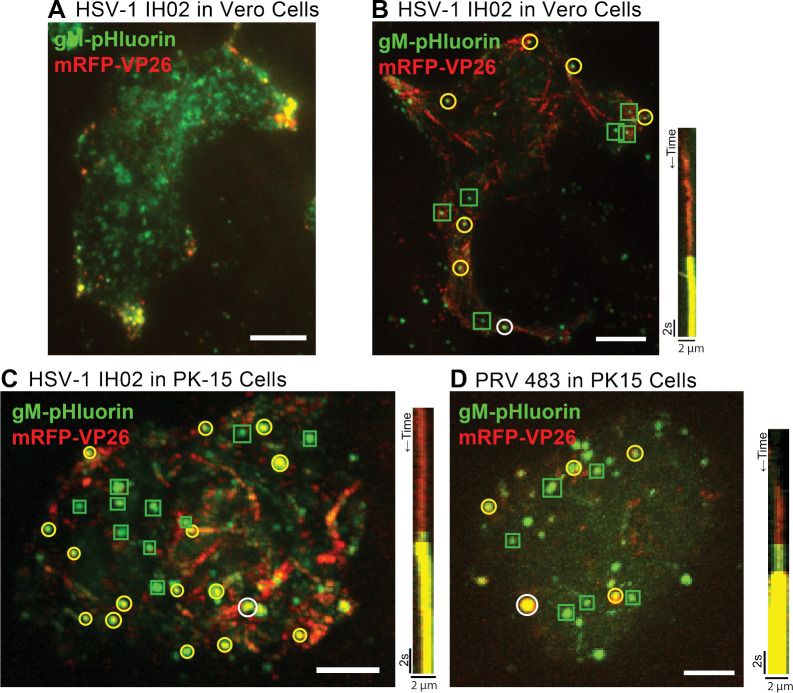
Single Virus Particle Exocytosis of HSV-1 and PRV **A.** HSV-1 IH02 infection in Vero cells, imaged by TIRF microscopy. Virus capsids (red) and gM-pHluorin (green) accumulate in clusters at the cell periphery at 6–7 hpi. **B-D** Maximum difference projections showing viral exocytosis events over time (left image). Green squares indicate exocytosis events containing gM-pHluorin (L-particles), yellow circles indicate exocytosis events containing both gM-pHluorin and mRFP-VP26 (virions), and white circles indicate the exocytosis events shown in the accompanying kymographs (right image). **B.** HSV-1 IH02 in Vero cells. Projection image represents 4:01 min:sec of imaging time at 6–7 hpi. **C.** HSV-1 IH02 in PK15 cells. Projection image represents 3:56 min:sec of imaging time at 6–7 hpi. **D.** PRV 483 in PK15 cells. Projection image represents 1:45 min:sec of imaging time at 4–5 hpi. In all panels, scale bars represent 10*μ*m.

To better understand how these large clusters of virus particles form, we infected Vero or PK15 cells with HSV-1 IH02, and imaged at 6–7 hpi. At this time point, HSV-1 IH02 particles were beginning to accumulate in clusters at the “corners” of Vero cells (Figure 3A). By tracking virus particles prior to exocytosis and observing the location of exocytosis, we found that viral exocytosis can occur outside of existing clusters, but that clusters appear to grow by individual particles undergoing exocytosis at or near these clusters (Figure 3A–B). Notably, the distribution of virion and L-particle exocytosis (as distinguished by the mRFP-VP26 capsid tag) was similar.

Because we previously reported no such accumulation of virus particles with PRV in PK15 cells [16, 17], we compared HSV-1 IH02 to PRV 483 in PK15 cells (Figure 3C–D). In contrast to Vero cells, there appears to be no large accumulations and no preferential sites of HSV-1 egress in PK15 cells (Figure 3C), similarly to PRV in PK15 cells (Figure 3D).

**Figure 4.**
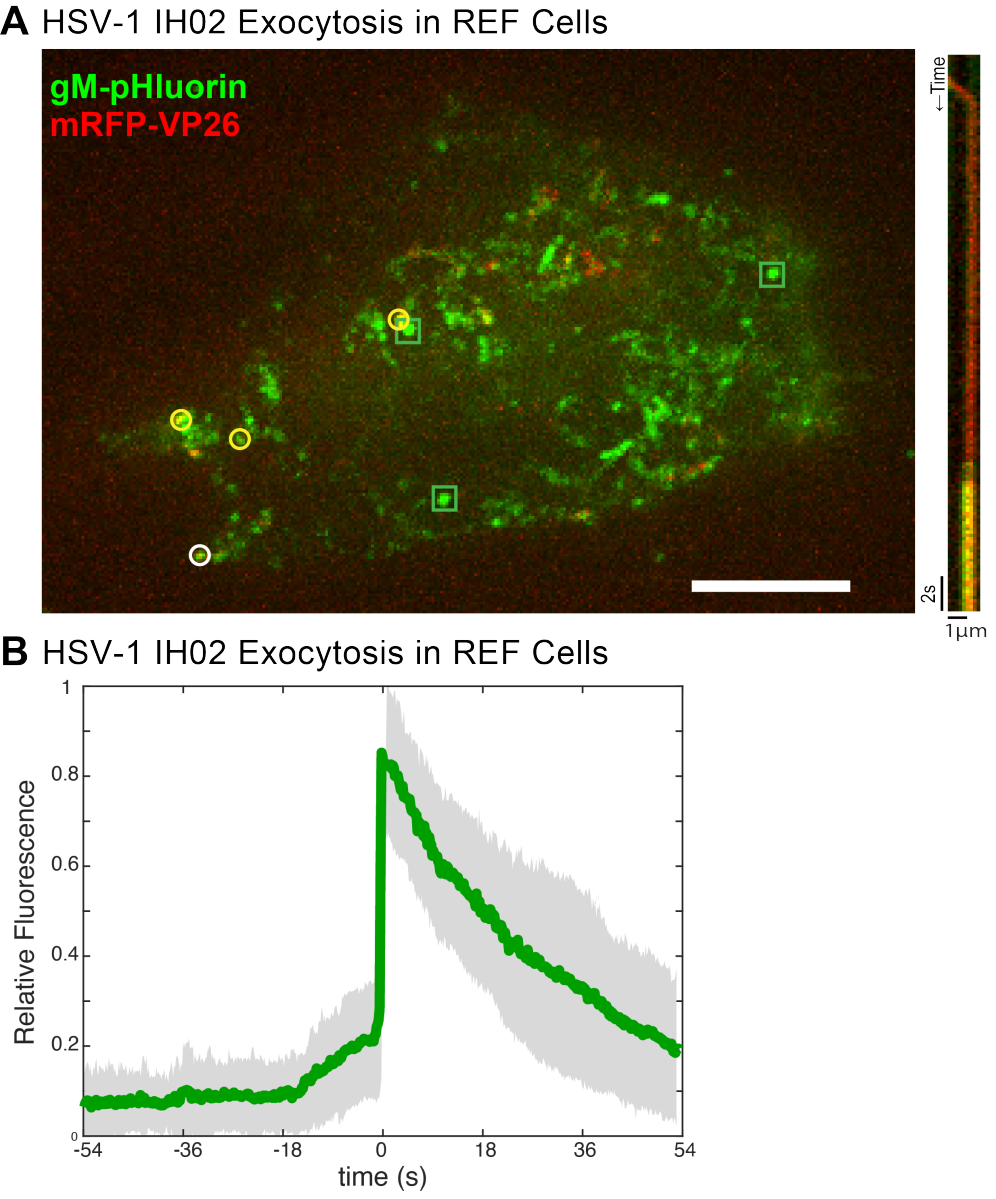
HSV-1 Exocytosis in Primary REF Cells **A.** Maximum difference projections showing viral exocytosis events over time (left image), of HSV-1 IH02 in REF cells. Green squares indicate exocytosis events containing gM-pHluorin (L-particles), yellow circles indicate exocytosis events containing both gM-pHluorin and mRFP-VP26 (virions), and white circle indicates the exocytosis event shown in the accompanying kymograph (right image). **B.** Relative fluorescence intensity of gM-pHluorin before, during, and after exocytosis of individual virus particles of HSV-1 IH02 in REF cells. Green line represents mean fluorescence and gray shading indicates standard deviation (n=42).

**Figure 5.**
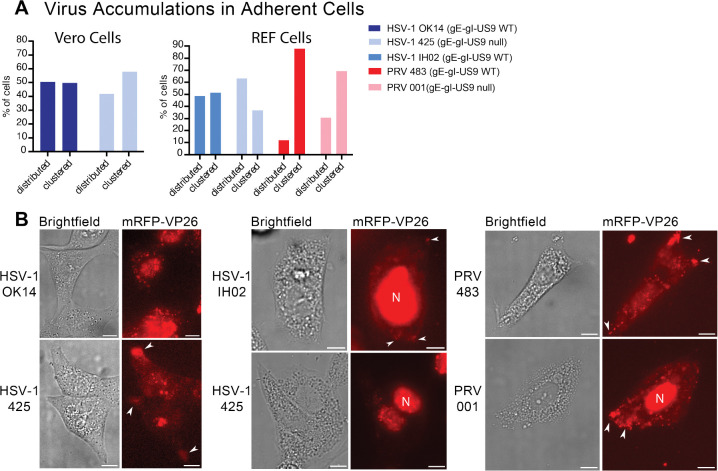
Accumulations of Progeny Virus Particles Accumulation of virus particles into clusters at the periphery of infected cells at 6–7 hpi. **A.**The percentage of cells (n=100) with clustered capsids was scored from random fields of view. **B.** Representative fluorescence microscopy images of infected Vero cells (right panels) and REF cells (center and left panels) showing peripheral clusters (arrowheads), or lack of clusters (no arrowheads) are provided. Scale bars = 15*μ*m. **A.** Accumulation/clustering of HSV-1 in Vero cells.

To determine whether this clustering occurs in a more biologically-relevant primary cell type, we prepared rat embryonic fibroblasts (REFs), and infected them with HSV-1 IH02 or PRV 483. In these cells, we observed HSV-1 exocytosis events (Figure 4A–B), similarly to Vero and PK15 cells. It was difficult to assess whether virus particles clustered to the same degree as in Vero cells, because REFs exhibited cell rounding and cytopathic effects earlier than in the transformed cell lines; however, clusters of virus particles were visible in ~50% of REF cells infected with HSV-1 IH02 and up to 90% of REF cells infected with PRV 483 (Figure 4A and 5A–B).

Altogether, these data show that the long-observed clustering of HSV-1 particles in Vero cells occurs due to preferential exocytosis of individual particles at these sites, rather than bulk release or post-exocytosis movements. Our prior observations, that this clustering does not occur with PRV in PK15 cells, is the result of cell type differences, not virus differences, as HSV-1 does not form clusters in PK15 cells (Figure 3C), and PRV does form clusters in REFs (Figure 5A–B).

### Viral Membrane Proteins gE, gI, and US9 are Not Required for Clustered Egress in Vero and REF Cells.

The three viral membrane proteins, gE, gI, and US9, have important functions in both HSV-1 and PRV egress. The clinical isolate HSV-1 MacIntyre and attenuated vaccine strain PRV Bartha, spread only in the retrograde direction and are incapable of anterograde spread in host nervous systems due mutations that disrupt the gE, gI, and US9 genes [42–47]. These proteins contribute to secondary envelopment, recruit microtubule motors for particle transport in multiple cell types [14, 48–50], and are required for axonal sorting and anterograde axonal spread in neurons [23, 42, 51–54]. However, it is not clear how mutations in gE, gI and US9 might affect egress in non-neuronal cells.

HSV-1 OK14, which is based on the 17syn^+^ laboratory strain, expresses functional gE, gI, and US9 [39, 55]. HSV-1 425 is based on the HSV-1 MacIntyre strain, which contains mutations that disrupt gE/gI/US9 function [46, 47]. Both viruses express an mRFP-VP26 capsid tag. At about 5 hpi, we manually categorized infected cells in random fields of view based on the presence of virus particle clusters at the cell periphery. Cells infected with HSV-1 425 demonstrated a roughly similar proportion of clustering compared to HSV-1 OK14 in Vero cells (Figure 5A). These results show that gE, gI, and US9 are not required for clustered egress of HSV-1.

To compare PRV to HSV-1, and further assess the function of gE, gI, and US9 in this clustering phenotype, we also infected cells with PRV recombinants. PRV 483 is based on the Becker laboratory strain, which expresses functional gE, gI, and US9 [56, 57] . We also constructed PRV 001, which is also based on PRV Becker, but contains the PRV Bartha deletion that removes the gE/gI/US9 genes. Both of these viruses express gM-pHluorin and mRFP-VP26. While PRV will infect and form plaques on Vero cells, we were unable to achieve sufficient levels of infection for our microscopy experiments - it is possible that the efficiency of plating of PRV in Vero cells is too low to achieve a high-MOI roughly synchronous infection in our experimental conditions. To overcome this limitation, we instead infected primary REF cells, which support robust infection of both viruses. HSV-1 and PRV exhibited clustering at the cell periphery, and gE, gI, and US9 proteins were not required for clustering. However, virus particle clustering appears to be slightly decreased in viruses that are deficient in gE, gI, and US9 (Figure 5A–B). These results further reinforce the idea that this clustering effect is common to both HSV-1 and PRV, and varies by cell type. However, the polarized trafficking that is mediated by gE/gI/US9 in neurons is not essential for clustered egress in these non-neuronal cells.

## Discussion

By producing an HSV-1 recombinant virus that expresses the pH-sensitive fluorescent protein, pHluorin, we have developed a live-cell microscopy assay that allows us to visualize the process of viral egress from infected cells. pHluorin is genetically fused to the viral envelope glycoprotein gM, is incorporated into virus particles, is quenched in the lumen of cellular vesicles, but dequenches upon exocytosis, allowing detection of virus particle exocytosis. We are able to detect individual virus particles undergoing exocytosis, and while this approach had been successful in previous alpha herpesvirus studies [16, 17], this is the first time that this approach has been applied to the important human pathogen, HSV-1.

Individual HSV-1 particles undergo exocytosis, not bulk release that has been observed with other herpesviruses [26]. The fact that virus particles have been observed in large clusters in infected cells [22, 23] is the result of individual particles undergoing exocytosis at preferential sites at the corners and edges of adherent cells, leading to the gradual accumulation of large clusters. However, this clustering does not occur in all cell types: both HSV-1 and PRV form clusters in Vero and primary REF cells, but not in PK15 cells.

Differences in intracellular transport and secretory mechanisms may explain why the spatial distribution of viral egress varies across these cell types. We, and others, have previously shown that alpha herpesvirus particles use cellular secretory pathways, regulated by Rab family GTPases, and recruit kinesin microtubule motors for intracellular transport to the site of exocytosis. Different cell types have been noted to express different kinesin motors and different Rab GTPases to transport and secrete cellular cargoes. As an extreme example of polarized trafficking and secretory pathways, in neurons, vesicles containing axonal cargoes can transport into the axon, but other vesicles are strongly excluded. The alpha herpesviruses appear to modulate axonal sorting and transport of viral secretory vesicles by recruiting additional kinesin motors via the viral gE, gI, and US9 proteins. However, in the present study, these viral factors are not required for transport to and exocytosis at preferential egress sites. It remains to be determined in future studies what cellular mechanisms are responsible for polarized transport and preferential exocytosis sites in these non-neuronal cells.

## Materials and Methods

### Cells.

Vero cells (ATCC, CCL-81), PK15 cells (ATCC, CCL-33), and primary rat embryonic fibroblasts (REFs) were all maintained in Dulbecco’s Modified Eagle’s Media (DMEM, Cytiva) supplemented with 10% FBS (Omega Scientific) and 1% penicillin-streptomycin (Hyclone), and incubated in a 5% CO_2_ incubator at 37°C.

REFs were collected from E16-17 Sprague-Dawley rat embryos (Charles River Laboratories), as follows: Animal work was performed in accordance with all applicable regulations and guidelines, and with the approval of the Institutional Animal Care and Use Committee (IACUC) at Arizona State University (protocol 20-1799R). The animal care and use program at Arizona State University has an assurance on file with the Office of Laboratory Animal Welfare (OLAW), is registered with the USDA, and is accredited by AAALAC International. Briefly, embryos were decapitated and internal organs removed. Remaining skin and connective tissue was trypsinized (Trypsin-EDTA, Gibco) at 37°C, pipetted vigorously in complete DMEM, and supernatants were plated onto 10cm cell culture dishes (Celltreat). REFs were passaged no more than 4 times before use in experiments [58].

### Viruses.

All HSV-1 or PRV strains were propagated and titered by plaque assay on Vero or PK15 cells, respectively, in DMEM supplemented with 2% FBS and 1% penicillin-streptomycin. HSV-1 17syn^+^ and OK14 were obtained from the Lynn Enquist laboratory (Princeton University) and verified by whole genome sequencing. HSV-1 OK14, which expresses an mRFP-VP26 capsid tag, was previously described [39]. HSV-1 425, which is based on HSV-1 MacIntyre and expresses an mRFP-VP26 capsid tag, was a kind gift from Esteban Engel (Princeton University) [47]. PRV 483 and PRV 495, which express gM-pHluorin and an mRFP-VP26 capsid tag, were previously described [16]. PRV BaBe was obtained from the Lynn Enquist laboratory (Princeton University) [56, 57].

### Construction of New HSV-1 Recombinants.

Three confluent 10 cm dishes of Vero cells were infected with HSV 17syn^+^ at MOI of 5 pfu/cell, and incubated overnight. Infected cells were rinsed with PBS, scraped from the dish, and lysed with an NP-40/Tris buffer (140mM NaCl, 2mM MgCl2, 0.5% Nonidet P-40, 200mM Tris). Nuclei were pelleted by centrifugation, and then lysed with 1% SDS in PBS. 100*μ*g/mL proteinase K was added, and incubated at 50°C for 1 hour. DNA was then isolated by phenol-chloroform extraction and ethanol precipitation. To produce HSV-1 IH01, a shuttle plasmid containing the pHluorin coding sequence flanked by HSV-1 sequences homologous to the HSV-1 UL10/gM locus was synthesized (Genewiz). This construct was designed to insert pHluorin into the first extravirion loop of the gM protein. Vero cells were cotransfected with linearized shuttle plasmid and DNA isolated from HSV-1 17syn^+^ infected cells using JetPrime transfection reagent (Polyplus). Following reconstitution of replicating virus, plaques were screened for expression of green fluorescence and plaque purified three times. To produce HSV-1 IH02, Vero cells were co-infected with HSV-1 IH01 and OK14, and progeny were screened for red and green fluorescence, and plaque purified three times.

### Construction of New PRV Recombinants.

PRV 001 was constructed by co-infecting PK15 cells with PRV 495 and PRV BaBe. PRV 495 expresses gM-pHluorin and mRFP-VP26, but also contains a deletion in the essential UL25 gene and cannot replicate on its own. PRV BaBe contains a deletion in the US region encoding gE, gI, and US9 [56, 57]. Following co-infection, progeny plaques were screened for green and red fluorescence. Several clones were picked, plaque purified three times, and further screened for lack of gE, gI, and US9 expression via western blot (data not shown).

### Fluorescence microscopy.

Vero, PK15, or REF cells were seeded at subconfluent density (~ 10^5^ cells/dish) on glass-bottom 35mm dishes (Celltreat, Ibidi, and Mattek), incubated overnight, and then infected with HSV-1 or PRV at a relatively high MOI (>1 pfu/cell). To account for differences in the efficiency of plating between different viruses and cells, the amount of inoculum needed to synchronously infect most cells was determined empirically by fluorescence microscopy. HSV-1 infected cells were imaged beginning at 5–6 hpi, and PRV infected cells were imaged beginning at 4–5 hpi, unless otherwise stated. Fluorescence microscopy was performed using a Nikon Eclipse Ti2-E inverted microscope in the Biodesign Imaging Core facility at Arizona State University. This microscope is equipped with TIRF and widefield illuminators, a Photometrics Prime95B sCMOS camera, a 60X high-NA TIRF objective, and objective and stage warmers for 37°C live-cell microscopy. For widefield fluorescence, a Lumencor SpectraX LED lightsource provided 470/24nm and 550/15nm excitation for green and red fluorescent proteins, respectively.

For TIRF microscopy, 488nm and 561nm lasers were used to excite green and red fluorescent proteins, respectively. Image analysis was performed using Fiji software [59]. Fluorescence microscopy images were prepared for publication using Adjust Brightness/Contrast, Reslice (to produce kymographs), and Plot Z-axis Profile (to measure fluorescence over time) functions in Fiji. Maximum difference projections were calculated as previously described [16], using the Duplicate, Stacks->Tools, Math->Subtract, and Z Project functions in Fiji. Maximum difference projection shows where fluorescence intensity increases most rapidly, which emphasizes exocytosis events and particle movement, and deemphasizes static features that do not change during the course of imaging. Ensemble averages of fluorescence intensity during exocytosis events (Figure 2B–C, Figure 4B) were calculated using Matlab (Mathworks).

### Western Blot.

Vero cells were infected with HSV-1 and PK15 cells were infected with PRV at high MOI and incubated overnight. Infected cells were lysed with an NP-40 lysis buffer (50mM Tris, 150mM NaCl, 1% Nonident P-40, diH2O) on ice for 3 minutes, nuclei were pelleted by centrifugation at 16000 rpm for 20 min, supernatants were mixed with 2X Laemmli sample buffer containing SDS and 2-mercaptoethanol, and heated to 95°C for 5 min. SDS-PAGE separation was run on Nu-Page precast gels (Invitrogen). Proteins were then transferred onto PVDF membrane (Immobilon-FL, Millipore) using a semi-dry transfer apparatus (BioRad). Membranes were blocked with a 5% nonfat dry milk solution, and probed with antibodies overnight. Mouse monoclonal anti-GFP antibody (Sigma) was used to detect pHluorin. Rabbit polyclonal antibodies targeting HSV-1 gM (PAS980), PRV gE, gI, and US9 were kindly provided by Lynn Enquist (Princeton University) [60, 61]. The next day, membranes were probed with fluorescent secondary antibodies (LI-COR) for one hour, washed, and imaged using a LI-COR Odyssey CLx scanner.

### Particle Imaging and pHluorin Quenching.

Vero cells in 35mm cell culture dishes were infected with HSV IH02 at high MOI. 24 hours post-infection, 100*μ*L of supernatant was pipetted onto glass bottom 35mm dishes for imaging. After allowing virus particles in the supernatant to adhere non-specifically to the glass, excess media was aspirated off and HBSS (Gibco) was added to prevent drying. Virus particles were subjected to a pH change by adding 15*μ*L PBS at pH 6. pH was then returned to neutral by adding an excess of PBS at pH 8. Imaging was performed using widefield LED illumination and 60X magnification to detect individual virus particles.

### Single Step Growth Curve and Plaque Size Measurements.

Vero cells were seeded to confluence in 35mm 6-well dishes and infected at MOI of 5 PFU/cell. The inoculated cells were incubated for 1 hour, washed with PBS three times, and incubated with viral medium at 37°C. At the specified time points, cells and supernatants were harvested. Mean titers were determined for each time point via serial dilution plaque assay in triplicate. Plaque sizes were measured in Fiji from brightfield and fluorescence microscopy images of 35mm wells of plaque assays from the single step growth curve.

## Importance

Alpha herpesviruses produce lifelong infections of their human and animal hosts. The majority of people in the world are infected with Herpes Simplex Virus 1 (HSV-1), which typically causes recurrent oral or genital lesions. However, HSV-1 can also spread to the central nervous system, causing severe encephalitis, and might also contribute to the development of neurodegenerative diseases. Many of the steps of how these viruses infect and replicate inside host cells are known in depth, but the final step, exiting from the infected cell, is not fully understood. In this study, we engineered a novel variant of HSV-1 that allows us to visualize how individual virus particles exit from infected cells.
